# Elucidation of the Mode of Action of a New Antibacterial Compound Active against *Staphylococcus aureus* and *Pseudomonas aeruginosa*

**DOI:** 10.1371/journal.pone.0155139

**Published:** 2016-05-11

**Authors:** Evelien Gerits, Eline Blommaert, Anna Lippell, Alex J. O’Neill, Bram Weytjens, Dries De Maeyer, Ana Carolina Fierro, Kathleen Marchal, Arnaud Marchand, Patrick Chaltin, Pieter Spincemaille, Katrijn De Brucker, Karin Thevissen, Bruno P. A. Cammue, Toon Swings, Veerle Liebens, Maarten Fauvart, Natalie Verstraeten, Jan Michiels

**Affiliations:** 1 Centre of Microbial and Plant Genetics, KU Leuven, Leuven, Belgium; 2 School of Molecular and Cellular Biology, University of Leeds, Leeds, United Kingdom; 3 Department of Information Technology (INTEC, iMINDS), U.Ghent, Ghent, Belgium; 4 Department of Plant Biotechnology and Bioinformatics, U.Ghent, Ghent, Belgium; 5 CISTIM Leuven vzw, Bio-Incubator, KU Leuven, Leuven, Belgium; 6 Centre for Drug Design and Discovery (CD3), Research and Development, KU Leuven, Leuven, Belgium; 7 Department of Laboratory Medicine, University Hospitals Leuven, Leuven, Belgium; 8 Department of Plant Systems Biology, VIB, Ghent, Belgium; 9 imec, Smart Systems and Emerging Technologies Unit, Department of Life Science Technologies, Leuven, Belgium; Purdue University, UNITED STATES

## Abstract

Nosocomial and community-acquired infections caused by multidrug resistant bacteria represent a major human health problem. Thus, there is an urgent need for the development of antibiotics with new modes of action. In this study, we investigated the antibacterial characteristics and mode of action of a new antimicrobial compound, SPI031 (N-alkylated 3, 6-dihalogenocarbazol 1-(sec-butylamino)-3-(3,6-dichloro-9H-carbazol-9-yl)propan-2-ol), which was previously identified in our group. This compound exhibits broad-spectrum antibacterial activity, including activity against the human pathogens *Staphylococcus aureus* and *Pseudomonas aeruginosa*. We found that SPI031 has rapid bactericidal activity (7-log reduction within 30 min at 4x MIC) and that the frequency of resistance development against SPI031 is low. To elucidate the mode of action of SPI031, we performed a macromolecular synthesis assay, which showed that SPI031 causes non-specific inhibition of macromolecular biosynthesis pathways. Liposome leakage and membrane permeability studies revealed that SPI031 rapidly exerts membrane damage, which is likely the primary cause of its antibacterial activity. These findings were supported by a mutational analysis of SPI031-resistant mutants, a transcriptome analysis and the identification of transposon mutants with altered sensitivity to the compound. In conclusion, our results show that SPI031 exerts its antimicrobial activity by causing membrane damage, making it an interesting starting point for the development of new antibacterial therapies.

## Introduction

Bacterial infections are among the most serious threats to human health. Millions of people acquire such infections each year, leading to increased mortality rates worldwide and an economic burden on society [1]. The Gram-positive pathogen *Staphylococcus aureus* is one of the major causes of nosocomial and community-acquired infections [2]. This bacterium accounts for 12.3% of all nosocomial infections in Europe, resulting in bacteremia and surgical wound infections, and is the main cause of implant-related infections [3,4]. Infections caused by community-acquired *S*. *aureus* can range from minor skin and tissue infections to progressive pneumonia [5]. The emergence of methicillin-resistant *S*. *aureus* has severely complicated the treatment of such infections. Currently, glycopeptide antibiotics such as vancomycin are often used to treat both methicillin-susceptible and methicillin-resistant infections [6,7]. However, in the past few years, there have been several reports of vancomycin-resistant *S*. *aureus* infections [8–10].

The Gram-negative bacterium *Pseudomonas aeruginosa* is another important pathogen that is frequently involved in nosocomial and community-acquired infections [11]. This pathogen is responsible for 8.9% of infections in European hospitals and is most commonly found in patients with cancer, cystic fibrosis or burn wounds [3,11]. In addition, *P*. *aeruginosa* is the main causative Gram-negative agent in implant-related infections [4]. Community-acquired infections caused by this pathogen include ulcerative keratitis, otitis externa and skin/soft tissue infections [11]. *P*. *aeruginosa* infections are usually treated with antibiotics such as β-lactams, aminoglycosides, or quinolones [12]. Alarmingly, multiple multidrug resistant strains of *P*. *aeruginosa* have emerged during the last years, some of them displaying resistance even towards the last-resort antibiotic polymyxin B [12,13].

**Fig 1:**
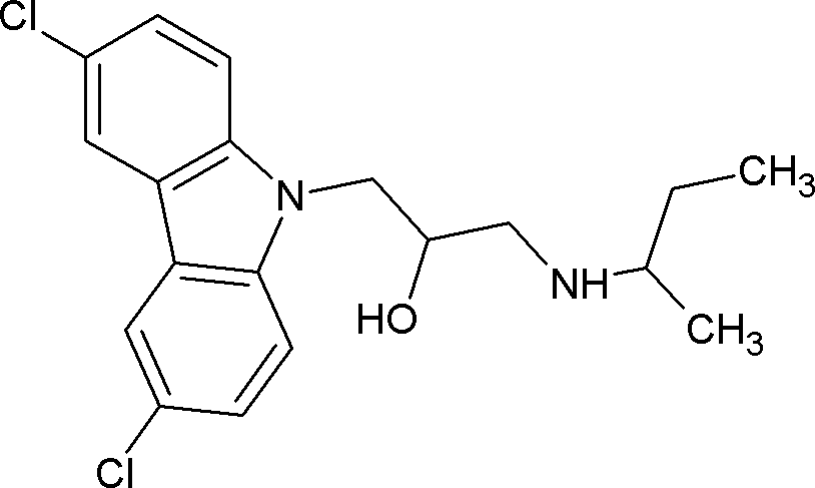
Structure of compound SPI031.

Lately, it has become clear that multidrug resistance is spreading at a very fast rate [1]. Unfortunately, only 5 new classes of antibiotics were marketed since 2000, and most of these do not work against Gram-negative pathogens [14]. Thus, there exists a pressing need for the development of new antibacterial agents. Recently, we identified a new antibacterial compound SPI031 (N-alkylated 3, 6-dihalogenocarbazol 1-(sec-butylamino)-3-(3,6-dichloro-9H-carbazol-9-yl)propan-2-ol) (Fig 1) with activity against a variety of bacteria, including *Escherichia coli* and the human pathogens *S*. *aureus*, *P*. *aeruginosa*, *Staphylococcus epidermidis* and *Porphyromonas gingivalis* [15]. In addition, we showed that the compound displays antifungal activity against *Candida albicans* [16]. Furthermore, we demonstrated that SPI031 has the clinical potential to be used as an antibacterial coating for implants, thereby reducing the incidence of implant-associated infections [17]. In the present study, we further investigated the antibacterial characteristics, including bactericidal activity and spontaneous resistance frequency, and mode of action of SPI031 using *S*. *aureus* and *P*. *aeruginosa* as model pathogens.

## Material and Methods

### Bacterial strains and chemicals

*S*. *aureus* SH1000 [18], *P*. *aeruginosa* PA14 [19], *Klebsiella pneumoniae* LMG 2095, *Acinetobacter baumannii* NCTC 13423, *Enterobacter aerogenes* LMG 2094, *Enterococcus faecium* LMG 8148 and *Staphylococcus aureus* ATCC 33591 were grown in 1/20 diluted trypticase soy broth (TSB, Becton Dickinson Benelux), TSB, lysogeny broth (LB) or on solid TSB medium containing 1.5% agar at 37°C.

SPI031 was supplied by CD3 (Leuven, Belgium) and stock solutions of 10 mM were prepared in dimethyl sulfoxide (DMSO). Vancomycin, polymyxin B, ofloxacin, tobramycin, ciprofloxacin, rifampicin, tetracycline, SDS, Triton X-100 and melittin were purchased from Sigma-Aldrich.

### Minimum Inhibitory Concentration (MIC) Assay

MIC values were determined in 1/20 TSB with a broth microdilution procedure as previously described [20]. All MIC values calculated in the present study are listed in S1 Table.

### Time-kill assay

*S*. *aureus* and *P*. *aeruginosa* cells were cultured to exponential phase at 37°C in 1/20 TSB and challenged with antibacterial compounds at 1x and 4x MIC (see S1 Table). At different time points (0, 0.1, 0.5, 1, 2, 3, 4, 5, 24 h), a 200 μl aliquot was removed from each sample, washed, serially diluted in MgSO4 (10 mM) and plated on TSB agar for enumeration of colony forming units (CFU).

### Spontaneous mutation frequency to resistance

The spontaneous mutation frequency of *S*. *aureus* and *P*. *aeruginosa* to resistance against different antibiotics was determined by plating bacterial inocula (10^7^−10^9^ CFU) on agar plates containing antibiotics at 5x MIC (see S1 Table). The number of viable cells present in the inoculum was determined by serial dilutions on drug-free agar plates. The mutation frequency was calculated by dividing the number of colonies formed on the plates after 48 h of incubation by the number of colonies on the drug-free plates. To confirm the results of this single-step mutation procedure, MIC tests were performed for all resistant mutants.

### Whole genome sequencing of spontaneous resistant mutants

Spontaneous SPI031-resistant mutants of *P*. *aeruginosa* were generated as described above and three independent mutants were selected for further analysis. Resistance of selected mutants was verified by MIC determinations. Subsequently, genomic DNA from *P*. *aeruginosa* wild-type (WT) and the spontaneous resistant mutants was isolated using the DNeasy Blood & Tissue Kit (Qiagen) following the manufacturer’s protocol. DNA quantity and purity were assessed using a NanoDrop ND-1000. Samples were sent to the Genomics Core Facility of EMBL (Heidelberg, Germany) for whole genome sequencing on an Illumina HiSeq 2000 platform. Assembly of the reads and further analysis were performed using the CLC Genomics Workbench program v8.0. Genome sequences of spontaneous resistance mutants were aligned with the genome sequence of the WT to detect mutations. A coverage above 10x and cutoff frequency of 75% were considered for the detection of the mutations. Detected mutations were verified by PCR amplification and Sanger sequencing.

### Macromolecular synthesis assay

Saturated cultures of *S*. *aureus* were diluted 1/100 in LB and grown to an OD_600_ of 0.2 at 37°C. with aeration. Cultures were labeled by the addition of either [methyl-^3^H]thymidine, [5,6-^3^H]uridine or L-[G-^3^H]glutamine at 1 μCi/ml to monitor synthesis of DNA, RNA, or proteins, respectively. After 10 min of incubation at 37°C, 100 μl of each culture was mixed with 100 μl of ice-cold 10% trichloroacetic acid (TCA) and stored on ice. The remainder of each culture was then treated with 4x MIC of SPI031, ciprofloxacin, rifampicin or tetracycline (see S1 Table). After 10 min of incubation, 100 μl of the treated cultures was mixed with an equal amount of 10% TCA and kept on ice for 30 min. TCA precipitates were collected under vacuum using a 96-well filter plate (96-well Unifilter GF/B, Perkin-Elmer), and filters onto which [5,6-^3^H]uridine-labelled samples had been deposited, were washed twice with 100 μl unlabeled uridine. Individual filters were then washed twice with 200 μl of 10% TCA each, and twice with 200 μl of acetic acid. Filter plates were dried, 25 μl scintillant (Microscint 20, Perking-Elmer) was added to each well, and radiolabeled incorporation was measured using a Chameleon multilabel plate scintillation counter (Hidex).

### Carboxyfluorescein leakage assay

Carboxyfluorescein (CF) loaded into liposomes matching the lipid composition of the cytoplasmic membrane of *S*. *aureus* were prepared as described previously [21]. Aliquots (5 μl) of the liposomes were mixed with 95 μl liposome buffer (10 mM HEPES, 107 mM NaCl, pH 7.4) containing SPI031 at a final concentration equivalent to 1x MIC and 4x MIC against *S*. *aureus* SH1000 (see S1 Table). Samples were incubated at 37°C while shaking and CF release (λ_ex_ = 485 nm, λ_em_ = 520 nm) was measured 10, 60 and 180 min after incubation using a FLUOstar omega plate reader (BMG Labtech). The percentage of liposome integrity after treatment was determined relative to the amount of CF release after treatment of the liposomes with 0.5% Triton X-100 (corresponding to 0% liposome integrity).

### Membrane permeability

SPI031-mediated permeabilization of the cytoplasmic membrane of both *S*. *aureus* and *P*. *aeruginosa* was measured using SYTOX green (Invitrogen, USA) as previously described [22], with some modification. Exponential-phase cells of *S*. *aureus* and *P*. *aeruginosa* grown in 1/20 TSB were washed and resuspended in phosphate-buffered saline to an OD_595_ of 0.5. Cells were stained with 1 μM of SYTOX green dye and treated with SPI031 (0x, 0.25x, 0.5x and 1x MIC; see S1 Table). After a period of incubation (30 min for *S*. *aureus*, 15 min for *P*. *aeruginosa*), fluorescence was measured (λ_ex_ = 504 nm, λ_em_ = 523 nm) using a Synergy MX multimode reader.

Outer membrane permeability of *P*. *aeruginosa* was assessed using the hydrophobic fluorescent probe 1-N-phenylnaphthylamine (NPN, Sigma, USA) as previously described [23], with minor modifications. Briefly, *P*. *aeruginosa* cells were grown to exponential phase in 1/20 TSB, washed and resuspended to an OD_595_ of 0.5 in buffer containing 5 mM HEPES, pH 7.2. Cells were mixed with NPN to a final concentration of 10 μM and treated with SPI031 (0x, 0.25x, 0.5x and 1x MIC; see S1 Table). Increase of fluorescence was measured immediately (λ_ex_ = 350 nm, λ_em_ = 420 nm) using a Synergy MX multimode reader (Biotek, Winooski, VT) at 37°C.

Fluorescence values were divided by corresponding OD_595_ values to correct for cell densities. In addition, values were corrected for background fluorescence by subtracting the values of the untreated control cultures stained with NPN or SYTOX green.

### Fluorescence microscopy

Exponential-phase cells of *S*. *aureus* and *P*. *aeruginosa* grown in 1/20 TSB were treated for 5 min with 0.5% DMSO (solvent control) or with 1x MIC of SPI031 (see S1 Table), centrifuged and stained with 10 μg/ml N-(3-triethylammoniumpropyl)-4-(p-diethylaminophenyl-hexatrienyl) pyridinium dibromide (FM 4–64, Molecular Probes). Samples were spotted on 2% agarose pads for imaging. Images were captured using a Zeiss Axio imager Z1 fluorescence microscope equipped with a EC Plan-Neofluar 100x objective, using the FM 4–64 channel (λ_ex_ = 540–580 nm; λ_em_ = 593–668 nm).

### RNA sequencing and data analysis

Overnight cultures of *P*. *aeruginosa* were diluted 1/100 in 1/20 TSB and were grown for 3 h until late-exponential phase. Next, cells were treated for 5 min with 0.2x MIC of SPI031 (see S1 Table) or 1% DMSO (solvent control). For each condition, three biological repeats were sampled. Total RNA isolation was performed as previously described [20]. Subsequently, rRNA was depleted using the Ribo-Zero rRNA Removal Kit (Illumina) as per manufacturer’s instructions. RNA integrity was analyzed using Experion RNA StdSens Chips (Bio-Rad). RNA quantity and purity was assessed using a NanoDrop ND-1000 spectrophotometer. Samples were sent to the Genomics Core Facility of EMBL (Heidelberg, Germany) for library construction and RNA sequencing using the Illumina HiSeq 2000 platform. The RNA sequencing data sets reported in this article are available in the ArrayExpress database (ArrayExpress accession: E-MTAB-4691; http://www.ebi.ac.uk/arrayexpress).

For data analysis, FastQC was used to verify the quality of the raw sequencing reads [24]. Next, sequences were aligned to the reference *P*. *aeruginosa* UCBPP-PA14 genome (NC_008463.1) with Bowtie2 using standard settings. Only samples in which more than 90% of the reads aligned exactly once were withheld for further analysis (see S2 Table). Subsequently, read counts per gene were calculated for each sample with Htseq-count [25]. The DESeq2 package [26] was used to normalize the data and to detect differential expression between treated and control samples using a False Discovery Rate (FDR) < 0.05. Next, a network analysis was carried out using PheNetic [27]. This analysis looks for both regulatory programs common to multiple differentially expressed genes and for downstream processes which are activated by differentially expressed genes, thus visualizing important molecular processes underlying the observed phenotype of the tested organism. Input data consist of three types of information: an interaction network of the tested organism, the differential expression data of all genes in the network and a gene list of significantly differentially expressed genes. Currently, no interaction network exists for *P*. *aeruginosa* PA14. For this reason, an interaction network for *P*. *aeruginosa* PAO1 was created, using different publicly available datasets (STRING [28], KEGG [29,30]). Subsequently, the genes of PA14 were mapped to the genes of PAO1 using the Rapid Annotation using Subsystem Technology (RAST) server [31]. Significantly differentially expressed genes were defined as the 100 genes having the highest log2-fold change. PheNetic was run using the standard parameters on the web server (22). Gene Ontology (GO) enrichment was performed on the resulting subnetwork using the Cytoscape [32] plugin BiNGO [33] (hypergeometric test, α < 0.05, FDR correction). GO annotations for *P*. *aeruginosa* PAO1 were retrieved from Uniprot [34].

### Screening of a transposon mutant library

A *P*. *aeruginosa* PA14 transposon library [35] was screened for increased sensitivity or resistance to SPI031. Mutants were allowed to grow in 1/20 TSB in 96-well plates. After overnight incubation, the cultures were diluted 100-fold in 1/20 TSB containing either 0.2x MIC or 2x MIC of SPI031 (see S1 Table) to detect hypersensitive or resistant mutants, respectively. Next, the plates were incubated while shaking for 24 h at 37°C. Growth was monitored by measuring the OD_595_. Mutants were identified as hypersensitive if their OD_595_ was less than 0.05 after incubation in the presence of 0.2x MIC of SPI031. On the other hand, mutants were identified as resistant if their OD_595_ was more than 0.1 after incubation in the presence of 2x MIC of SPI031. Subsequently, the susceptibility of the selected mutants was validated by detailed monitoring of growth in the presence of either 0.2x or 2x MIC of SPI031 using an automated OD plate reader (Bioscreen C, Oy Growth curves Ab Ltd).

### Statistical analysis

All experiments were repeated independently at least 3 times. Statistical significance of data was determined by applying a student’s t-test using GraphPad Prism version 5 (GraphPad Software, USA). Differences were considered significant if *p ≤ 0.05.

## Results

### SPI031 displays rapid killing activity against pathogenic bacteria

**Fig 2:**
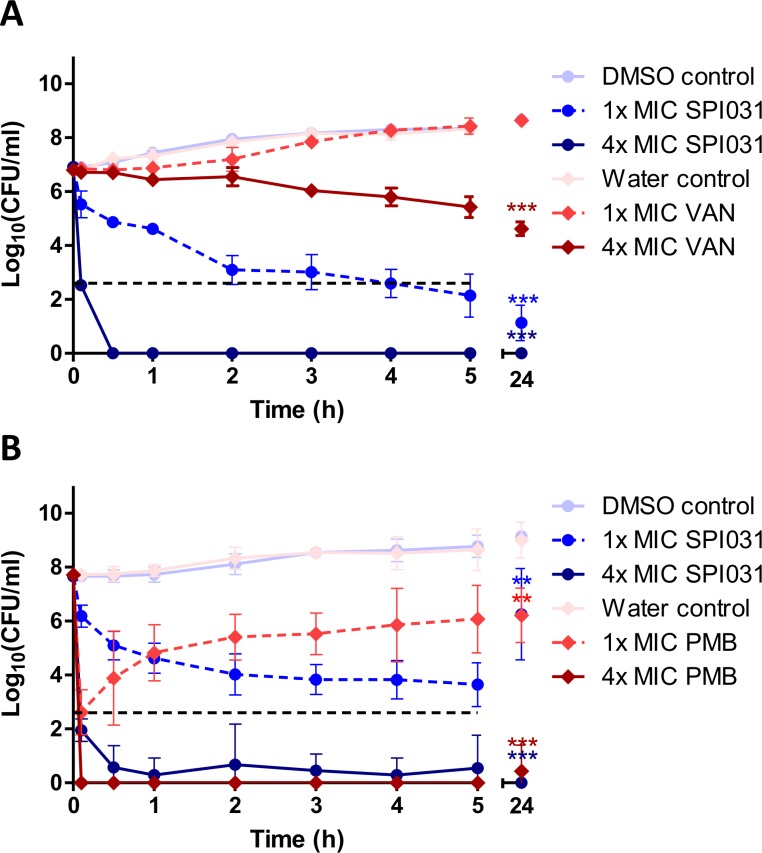
Time-kill kinetics of SPI031 against *S*. *aureus* and *P*. *aeruginosa*. (A) Concentration-dependent killing of *S*. *aureus* by SPI031 and vancomycin (VAN). (B) Concentration-dependent killing of *P*. *aeruginosa* by SPI031 and polymyxin B (PMB). All data represent means ± standard error of the mean (SEM) from 3 independent experiments (*p < 0.05; **p < 0.01; ***p < 0.001). The black dotted lines indicate the lower limit of detection.

Recently, we demonstrated that SPI031 exhibits antibacterial effects against different bacterial pathogens (MIC between 4.63 and 18.5 μg/ml) [15]. In the present study, time-kill assays were performed to analyze the killing rate of SPI031 and to compare it with that of conventional antibiotics frequently used in clinical settings. MIC values for SPI031 and selected antibiotics were determined and are listed in S1 Table. Fig 2A shows the killing curves of SPI031 and vancomycin for *S*. *aureus*. At 1x MIC of SPI031, the bactericidal endpoint was achieved after 24 h, with a reduction of CFU by almost 6 log units. Vancomycin had a low bactericidal activity at 1x MIC and substantial regrowth was observed after 2 h. At 4x MIC of SPI031, more than 99% of the bacteria were killed within 30 min. In comparison, the killing activity of vancomycin at 4x MIC was much slower, with a reduction of the initial inoculum by only 2 log units within 24 h.

The killing kinetics of SPI031 and polymyxin B against *P*. *aeruginosa* are shown in Fig 2B. At 1x MIC of SPI031, a 4-log reduction of viable counts was achieved and regrowth was observed after 24 h. At 1x MIC of polymyxin B, a 5-log reduction of the bacterial inoculum was obtained within 10 min, but fast regrowth was observed after 30 min. At 4x MIC, the bactericidal activity of both SPI031 and polymyxin B was very fast (7-log reduction within 30 min), indicating that at higher therapeutic concentrations, SPI031 and polymyxin B display similar killing activities.

### Frequency of resistance development against SPI031 is low

To investigate the potency of spontaneous resistance development against SPI031, the frequency by which *P*. *aeruginosa* mutants resistant to SPI031 emerge was determined, and compared to those associated with conventional antibiotics. The mutation frequency of *P*. *aeruginosa* for SPI031 (6.09 ± 2.10 x 10^−8^) was significantly lower than for polymyxin B (2.20 ± 0.44 x 10^−7^) and not significantly different than that for ofloxacin (4.03 ± 1.10 x 10^−8^). Interestingly, no spontaneous SPI031-resistant mutants of *S*. *aureus* could be generated (limit of detection < 10^−9^).

### SPI031 exhibits non-specific inhibition of macromolecular biosynthesis pathways

**Fig 3:**
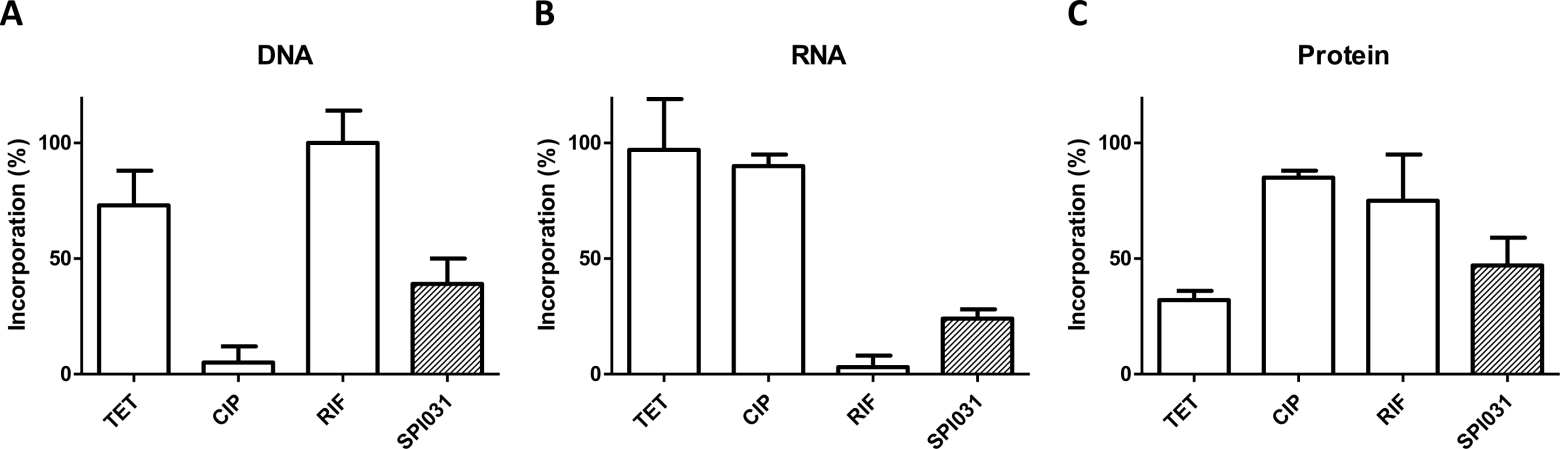
Effect of SPI031 on macromolecular synthesis in *S*. *aureus*. Incorporation of [methyl-^3^H]thymidine (A), [5,6-^3^H]uridine (B) and L-[G-^3^H]glutamine (C) by *S*. *aureus* after treatment with SPI031, ciprofloxacin, rifampicin or tetracycline at 4x MIC. Incorporation was expressed as percentage of untreated control. Values shown are means ± SD of triplicate determinations.

To investigate the effect of SPI031 on macromolecular pathways, a macromolecular synthesis assay was performed. The percentage of incorporation of radiolabeled precursors into macromolecules was determined upon exposure to SPI031 and was compared with results obtained after treatment with known inhibitors of DNA, RNA or protein synthesis: ciprofloxacin, rifampicin and tetracycline, respectively (Fig 3). At 4x MIC of SPI031, no preferential inhibition of tested macromolecular processes was observed, a profile that is observed for antibacterial agents that act by disrupting the integrity of the cytoplasmic membrane [36–39].

### SPI031 permeabilizes the membrane(s) of both *S*. *aureus* and *P*. *aeruginosa*

**Fig 4:**
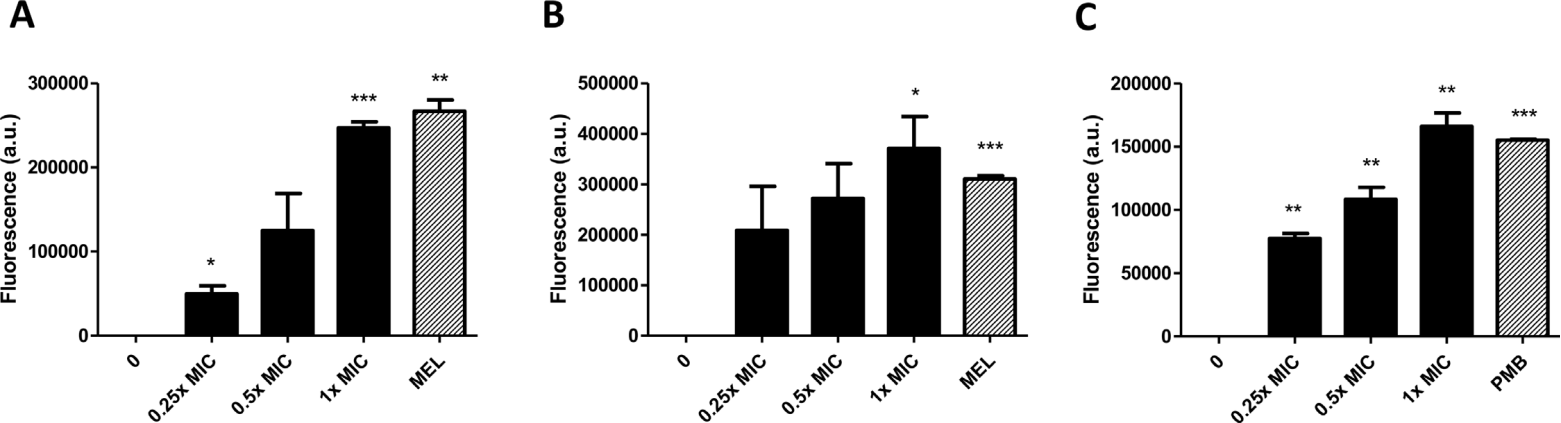
Effect of SPI031 on membrane permeability. (A) Effect of increasing concentrations of SPI031 on the membrane permeability of *S*. *aureus*, monitored by the uptake of SYTOX green. Cells treated with melittin (MEL) (1x MIC) served as a positive control. (B) Inner membrane permeabilization of *P*. *aeruginosa* after treatment with different concentrations of SPI031, determined by measuring SYTOX green uptake. Melittin (MEL) (1x MIC) was used as a positive control. (C) Outer membrane permeabilization of *P*. *aeruginosa* after treatment with different concentrations of SPI031, assessed by quantifying NPN uptake. Cells treated with polymyxin B (PMB) (1x MIC) were used as a positive control. Data represent the means of three independent replicates ± SEM (*p < 0.05; **p < 0.01; ***p < 0.001 compared to untreated control).

Based on the results of the macromolecular synthesis analysis, we further investigated membrane permeabilisation. Permeabilization of the membrane of *S*. *aureus* after treatment with SPI031 was assessed using the nucleic acid stain SYTOX green. This stain only enters the cytoplasm if the membrane is compromised. Upon uptake, SYTOX green binds to nucleic acids, which in turn results in increased fluorescence [40]. Addition of SPI031 to *S*. *aureus* cells caused a concentration-dependent increase in SYTOX green fluorescence, indicating that SPI031 permeabilizes the membrane (Fig 4A). At 1x MIC of SPI031, membrane permeabilization was comparable to that after treatment with the toxin melittin from bee-venom, which was used as a positive control. Inner membrane permeability of *P*. *aeruginosa* after treatment with SPI031 was also investigated using SYTOX green (Fig 4B). SPI031 was able to rapidly induce SYTOX green uptake, suggesting that the compound also permeabilizes the inner membrane of *P*. *aeruginosa*.

Next, permeabilization of the outer membrane of *P*. *aeruginosa* was determined after treatment with different concentrations of SPI031, using the hydrophobic probe NPN. This probe is normally excluded from bacterial membranes due to the presence of lipopolysaccharide molecules on the outer membrane [41]. However, when the outer membrane is permeabilized, NPN can enter the membranes, leading to a strong increase in fluorescence [42]. As shown in Fig 4C, SPI031 rapidly permeabilized the outer membrane of *P*. *aeruginosa* in a concentration-dependent manner. At 1x MIC of SPI031, the NPN uptake was similar to that of the positive control polymyxin B.

### SPI031 targets the phospholipid component of bacterial membranes

**Fig 5:**
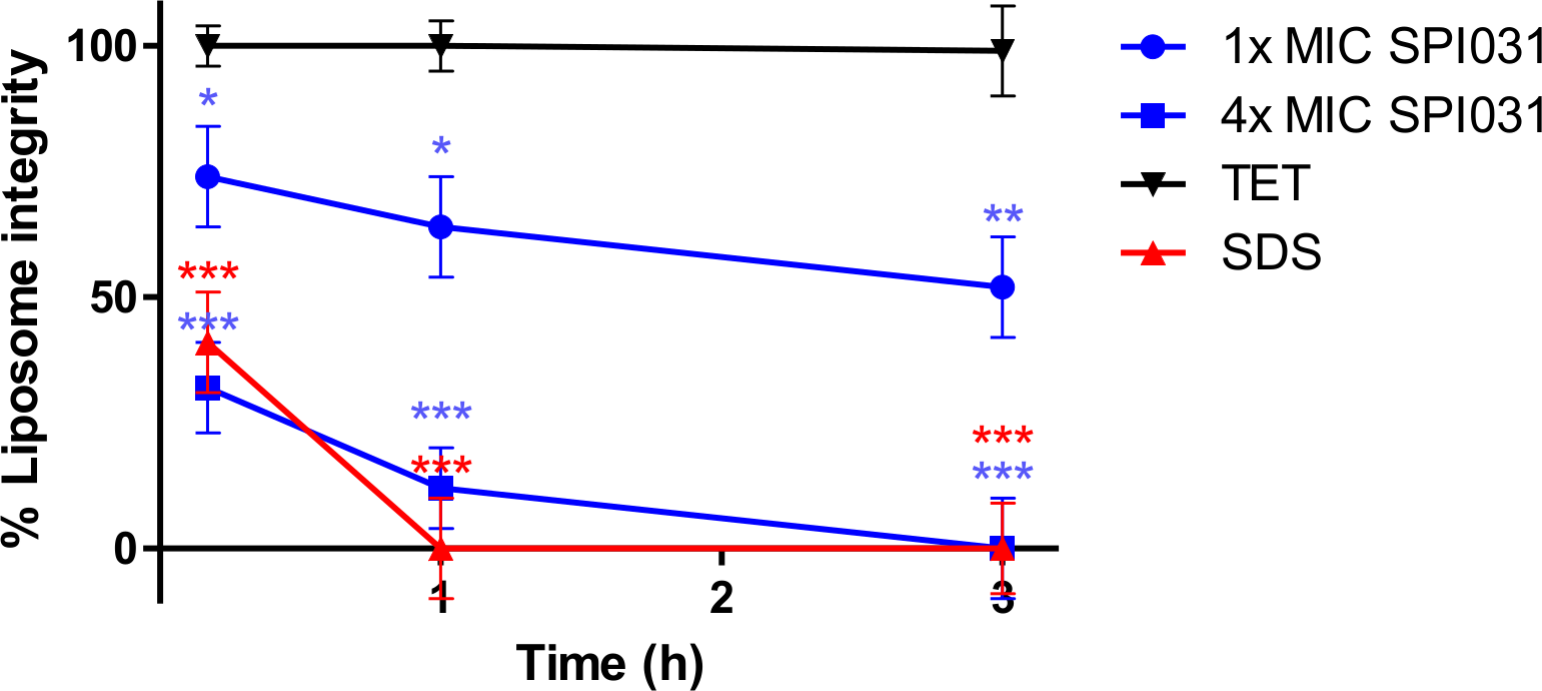
Integrity of *S*. *aureus* liposomes after treatment with SPI031 at 1x MIC and 4x MIC. SDS (5%) and tetracycline (TET) (4x MIC) were used as positive and negative controls, respectively. Means and standard deviation (SD) of 3 independent experiments are shown (*p < 0.05; **p < 0.01; ***p < 0.001 compared to treatment with tetracycline).

In addition to lipids, the bacterial membrane contains many other biological molecules. To investigate the direct interaction of SPI031 with the phospholipid bilayer, a carboxyfluorescein (CF) leakage assay was performed. For this assay, CF-loaded liposomes were used that mimic the composition of the phospholipid bilayer of *S*. *aureus*. As shown in Fig 5, SPI031 caused substantial release of CF at 4x MIC, and an intermediate release at 1x MIC. These results indicate that SPI031 is able to interact with the phospholipid bilayer, which in turn results in destabilization of the cytoplasmic membrane.

### Membrane damage caused by SPI031 can be visualized microscopically

**Fig 6:**
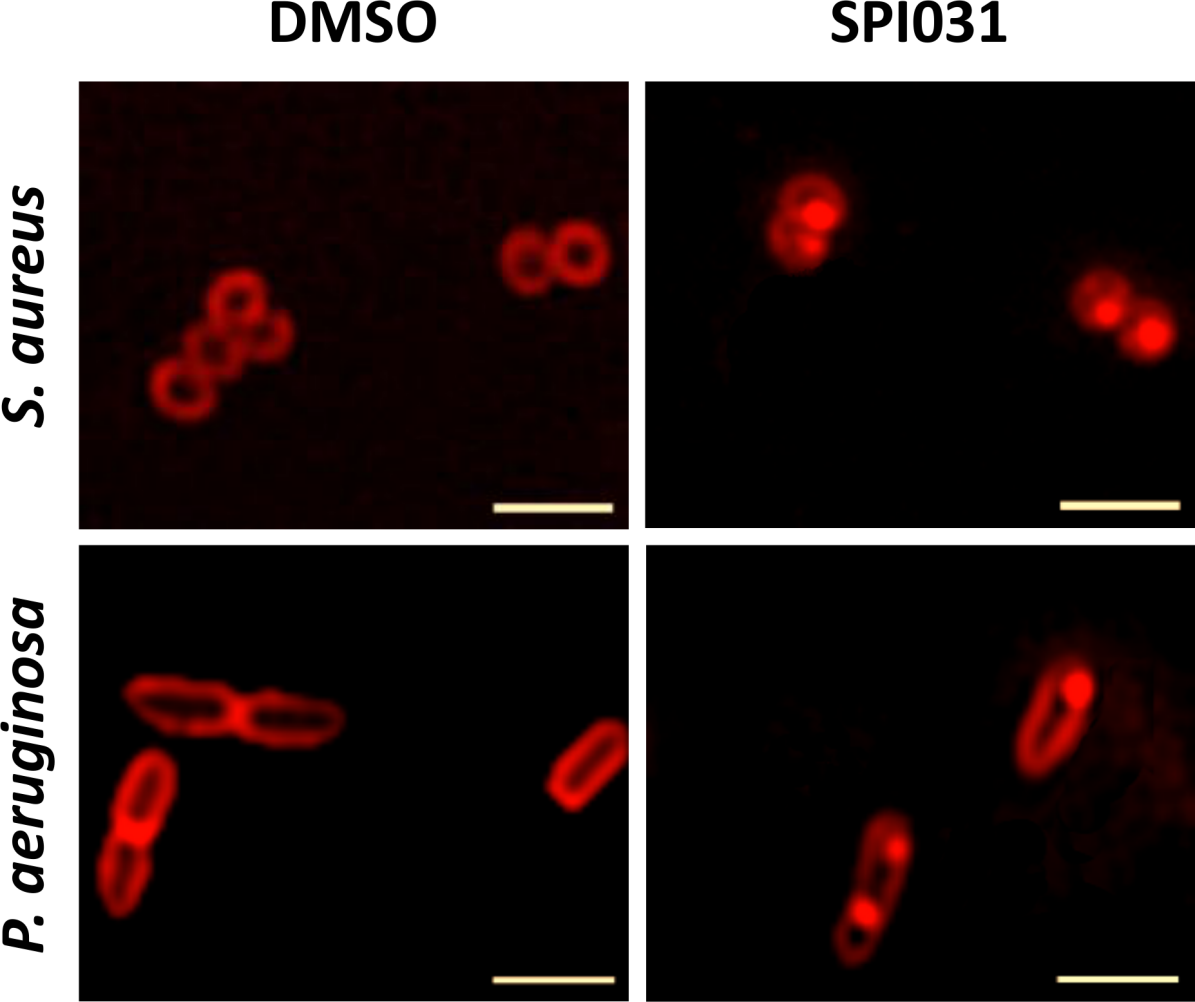
Microscopic analysis of the cell membrane after treatment with SPI031. Fluorescent images of *S*. *aureus* cells (upper row) and *P*. *aeruginosa* cells (lower row) stained with FM 4–64 in the absence or presence of 1x MIC of SPI031. Scale bar corresponds to 2 μm. Images were processed with unsharp mask of Zen 2.0.

To further investigate the effect of SPI031 on the membrane, the membrane stain FM 4–64 was used (Fig 6). Treatment of exponentially growing cells of *S*. *aureus* and *P*. *aeruginosa* with solvent control DMSO resulted in uniformly stained membranes. In contrast, treatment of the cells with SPI031 at 1x MIC resulted in specific membrane accumulations. Heterogeneity in membrane staining was also seen in *P*. *aerugino*sa cells treated with 1x MIC of polymyxin B. In contrast, *P*. *aeruginosa* cells exposed to 1x MIC of tobramycin showed no membrane defects (data not shown). Overall, these results further corroborate a clear direct effect of SPI031 on the bacterial membrane.

### Whole genome sequencing of SPI031-resistant mutants reveals mutations in genes coding for proteins involved in outer membrane synthesis and efflux mechanisms

Mutational analysis of spontaneous resistant mutants is an approach commonly used to gain more information about primary targets of new antibacterial compounds [43–46]. Therefore, three independent spontaneous SPI031-resistant mutants of *P*. *aeruginosa* were selected and were subjected to whole genome sequencing. All identified mutations are listed in S3 Table. One mutant carried mutations in *nfxB*, which codes for the repressor of the MexCD-OprJ multidrug efflux pump [47]. These findings indicate that SPI031 may be a substrate for this efflux pump. The two other mutants had mutations in genes coding for proteins involved in outer membrane synthesis (*htrB* and PA14_23400) [48,49]. For these mutants, we hypothesize that a change in the structure of the outer membrane is responsible for the observed resistance. In addition, two *P*. *aeruginosa* mutants acquired mutations in intergenic regions.

### Transcriptome analysis of bacteria challenged with SPI031 reveals altered expression of genes involved in fatty acid biosynthesis and lipid metabolic processes

**Fig 7:**
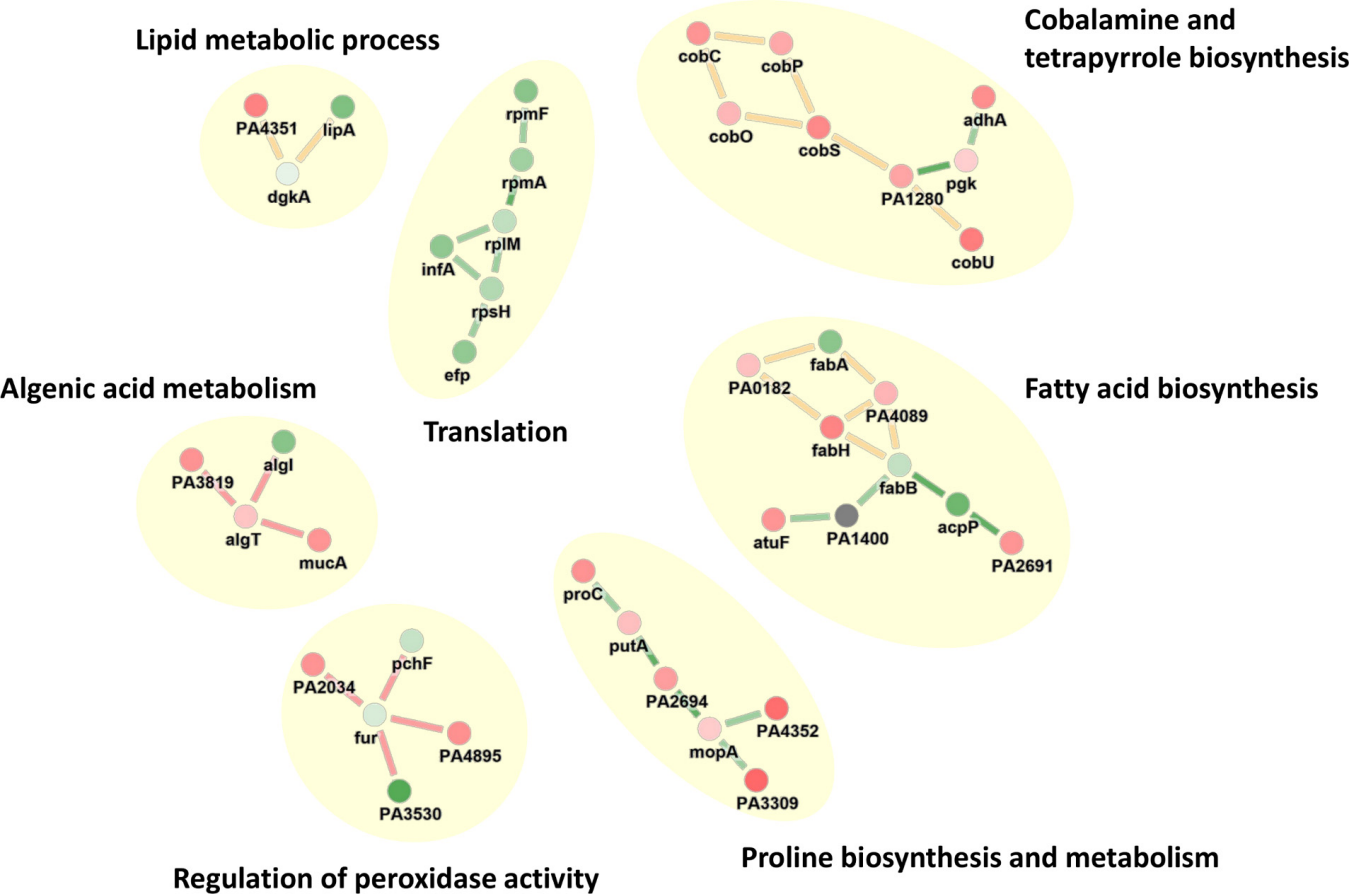
Network analysis of differential expression data. Resulting subnetwork from network analysis of RNAseq data using PheNetic [27]. This subnetwork shows molecular mechanisms which are differentially active when comparing *P*. *aeruginosa* cells treated with 0.2x MIC of SPI031 to *P*. *aeruginosa* cells treated with DMSO. Nodes and connecting lines represent genes and interactions between these genes, respectively. Red dots represent genes overexpressed in the SPI031-treated organisms with respect to the DMSO-treated organism and vice versa for green dots. The color of the connecting lines represents the type of interaction in between genes. Yellow lines represent metabolic interactions, red lines represent regulatory interactions and green lines represent protein-protein interactions.

A transcriptome analysis was performed using RNA sequencing to uncover the cellular responses to SPI031. To assess the direct effects of the compound on cellular processes, the transcription profile was determined from cells treated for 5 min with a sub-inhibitory concentration of SPI031. 304 genes were differentially expressed upon treatment with SPI031. Subsequently, a network analysis was conducted using PheNetic (Fig 7). To investigate the regulatory pathways altered by SPI031, a GO enrichment analysis was performed on the resulting subnetwork (see S4 Table). This analysis demonstrated that SPI031 alters expression of genes involved in fatty acid biosynthesis and lipid metabolic processes, indicating that SPI031 induces membrane damage. In addition, SPI031 also affects expression of genes involved in alginic acid metabolism, peroxidase activity, cobalamine and tetrapyrrole metabolism, translation and proline biosynthesis and metabolism, which are believed to be secondary effects.

### Mutants with aberrant cell division show increased sensitivity to SPI031

To uncover tolerance mechanisms of *P*. *aeruginosa* against SPI031 and to obtain more insight into its mode of action, a library of transposon insertion mutants of *P*. *aeruginosa* [35] was screened to identify genes associated with altered sensitivity to the compound. This led to the recovery of two SPI031-hypersensitive mutants affected in *minC* and *minD*, which are genes encoding proteins that are involved in cell division (see S1 Fig). More specifically, MinC and MinD act in concert to form a nonspecific inhibitor of septation [50]. Hypersensitivity to SPI031 of an *E*. *coli minC* deletion mutant [51] could also be demonstrated (data not shown). These results suggest that an aberrant cell division results in increased sensitivity to the antibacterial activity of SPI031.

## Discussion

Recently, we identified the promising antibacterial compound SPI031, which possesses antibacterial activity against various Gram-positive and Gram-negative pathogens, including the clinically important pathogens *S*. *aureus* and *P*. *aeruginosa* [15]. Moreover, additional tests demonstrated antibacterial activity against a representative selection of ESKAPE pathogens (see S5 Table). Furthermore, we demonstrated that SPI031-functionalised titanium surfaces show significant antibacterial activity both *in vitro* and *in vivo* [17].

In the present study, time-kill kinetic studies were performed to assess the bactericidal activity of SPI031 against *S*. *aureus* and *P*. *aeruginosa*. Interestingly, SPI031 displayed superior bactericidal activity against *S*. *aureus* compared to vancomycin, an antibiotic frequently used to treat staphylococcal infections [6,7]. It should be noted that time-kill assays were performed with late exponential phase populations of *S*. *aureus*, which could explain the observed low activity of vancomycin [52,53]. When comparing the killing kinetics of SPI031 and last-resort antibiotic polymyxin B for *P*. *aeruginosa*, regrowth was observed for both antibacterial agents at 1x MIC, although this occurred at a much slower rate for SPI031. The fast regrowth observed after treatment with low concentrations of polymyxin B could be explained by selective amplification of less-susceptible sub-populations [54] and is in accordance with previous studies examining the time-kill kinetics of polymyxins [55,56]. At higher concentrations, both antibiotics rapidly displayed bactericidal activities against *P*. *aeruginosa*, which is a desirable feature in the fight against recalcitrant infections. In follow-up studies, it will be investigated whether these *in vitro* time-kill data are predictive of *in vivo* efficacy.

In order to be effective in treatment, it is important that the incidence of resistance to SPI031 is low. For this reason we determined the frequency by which *P*. *aeruginosa* mutants resistant to SPI031 emerge. The results were compared to those of conventional antibiotics. The mutation frequency of *P*. *aeruginosa* for SPI031 and ofloxacin was lower compared with polymyxin B. In addition, no spontaneous SPI031-resistant mutants of *S*. *aureus* could be recovered. These results highlight the potential advantage of using SPI031 in the treatment of infections.

To gain more insight into the mode of action of SPI031, we monitored the ability of the compound to inhibit bacterial macromolecular synthesis processes. No specific inhibition of DNA, RNA, or protein synthesis could be observed. These results are reminiscent of those obtained after treatment of bacterial cells with membrane damaging agents that cause leakage of cytoplasmic components [36–39], suggesting that SPI031 has membrane damaging activity. It is also known that membrane damaging agents have a rapid bactericidal activity and a low potential for the development of resistant mutants [57], which is consistent with the results discussed above.

To further investigate the effect of SPI031 on the cell membrane, permeability assays were performed. SPI031 was able to disrupt the membrane of *S*. *aureus*, facilitating the intracellular uptake of SYTOX green, in a dose-dependent manner. SPI031 also permeabilized the inner and outer membrane of *P*. *aeruginosa*, as evidenced by the uptake of the fluorescent probes SYTOX green and NPN, respectively. Furthermore, SPI031 was able to induce CF leakage from liposomes, suggesting that the compound causes membrane permeabilization through direct physical interaction with the phospholipid bilayer. Also, by using the fluorescent membrane dye FM4-64, it was shown that *S*. *aureus* and *P*. *aeruginosa* cells treated with SPI031 have distinct membrane accumulations, which is in corroboration with the results obtained in the biophysical studies mentioned above.

Whole genome sequencing of spontaneous SPI031-resistant mutants of *P*. *aeruginosa* revealed mutations in *nfxB*, which codes for the repressor of the MexCD-OprJ multidrug efflux pump. These mutations probably cause a loss of function of NfxB, which leads to up-regulation of *mexCD-oprJ* and increased resistance [58]. These results confirm our previous observations regarding the role of the *mex* efflux pumps in intrinsic resistance of *P*. *aeruginosa* for SPI031 [15]. These findings are not unexpected given that practically all classes of antibiotics are known to be substrates of multidrug efflux pumps [59]. In addition, mutations were found in *htrB* and PA14_23400, two genes that are involved in outer membrane synthesis. HrtB is an acyltransferase involved in lipid A biosynthesis and PA14_23400 presumably plays a role in O-antigen biosynthesis [48,49]. We assume that these mutations confer alterations in outer membrane composition, thereby leading to increased resistance. Hence these results may indicate a direct effect of SPI031 on the outer membrane, as was observed experimentally.

Transcriptome analysis demonstrated that the expression of genes involved in the fatty acid biosynthesis and lipid metabolic processes was already altered after 5 min of treatment with SPI031. We hypothesize that the cell tries to compensate for the membrane defects induced by SPI031, which supports the results obtained above. In addition, SPI031 also affects the expression of genes involved in alginic acid metabolism. Alginate is an exopolysaccharide that plays a central role in the formation of biofilms [60]. Thus, these results may suggest that SPI031 inhibits biofilm formation by interfering with alginate production. This is in line with our previous observations which showed that SPI031 exhibits antibiofilm properties [15]. Furthermore, the analysis showed an enrichment of genes related to peroxidase activity, which is a component of the oxidative stress response. Interestingly, SPI031 also interferes with the transcription of genes that are required for cobalamine and tetrapyrrole metabolism. To our knowledge, these metabolisms have never been linked before to the mode of action of an antibiotic agent. Thus, further research is needed to clarify these findings.

Finally, we found that *minC* and *minD* transposon mutants displaying aberrant cell division are hypersensitive to SPI031. These results suggest that cell division contributes to tolerance mechanisms against SPI031 and are in agreement with a previous study, showing that a mutant lacking a gene that regulates septum formation is more susceptible to a membrane-targeting antibiotic [61]. At this stage, it is not clear how exactly aberrant cell division leads to increased susceptibility to SPI031. Changes in the divisome might induce destabilization of the membrane(s), facilitating the direct interaction of SPI031 with the membrane. The role of cell division in tolerance mechanisms against SPI031 will be studied further in the near future. As antibiotic-resistant bacteria are spreading at an alarming rate, the development of alternative treatment options becomes crucial. One of these approaches involves the identification of compounds that potentiate the activity of an antibiotic, thereby lowering the risk for development of resistance [62]. Hence, in future experiments, it could be interesting to assess whether inhibitors of cell division potentiate the activity of SPI031.

Per mode of action, SPI031 could potentially be used in antibacterial treatments. However, when tested against human keratinocytes and human hepatoma cells, a reduction in cell viability was observed even at low concentrations (data not shown). Toxicity is often a major obstacle in therapeutic application of membrane-damaging antibacterial agents. The design of structural analogs of SPI031 with higher specificity towards the bacterial membranes and minimal toxicity towards eukaryotic membranes may provide a solution to this problem. Indeed, previous work has shown that the evaluation of structural analogs of antibacterial molecules represents a good strategy to improve antibacterial potency and to minimize toxicity [63–65]. Of note, various carbazoles have been reported that exhibit low cellular toxicity at clinically relevant concentrations, pointing to the possibility to further develop SPI031 into a new agent for the treatment of topical bacterial infections [16]. Furthermore, we previously demonstrated that SPI031 is a suitable candidate for the development of new anti-infective coatings on implants [17]. In that study, we developed titanium substrates on which SPI031 was covalently linked. We found that immobilized SPI031 remains active under both *in vivo* and *in vivo* conditions. In addition, we demonstrated that immobilized SPI031 displays no cytotoxic effects on osteogenic and vasculogenic human cells important for osseointegration and bone repair.

In summary, we have shown that SPI031 displays rapid bactericidal activity and a low tendency for the development of resistance. In addition, we have demonstrated that the membrane is the primary target of SPI031. In recent years it has become clear that membrane-targeting agents are excellent candidates for the treatment of persistent infections caused by biofilm-forming bacteria [57]. Taken all these factors into account, we believe that SPI031 has the potential to serve a as backbone molecule for the development of new anti-infective therapies, such as coatings for implants. Further investigations such as physicochemical studies, toxicity tests, animal efficacy and pharmacokinetic/pharmacodynamic studies will be needed to fully reveal the therapeutic potential of this class of molecules.

## Supporting Information

S1 Fig Hypersensitivity of *P*. *aeruginosa minC* and *minD* mutants to SPI031. Growth curves of WT, *minC* mutant and *minD* mutant in the absence and presence of 0.2x MIC of SPI031. OD_595_ was measured every 10 min using an automated OD plate reader. The experiment was repeated three times and one representative repeat is shown.
(TIF)

S1 Table MIC values of SPI031 and other antibiotics against *S*. *aureus* and *P*. *aeruginosa*. (DOC)

S2 Table Bowtie2 alignment statistics for samples treated with DMSO (solvent control) and 0.2x MIC of SPI031. (DOC)

S3 Table Mutations identified in spontaneous SPI031-resistant mutants^1^. ^1^Abbreviations: SRM, spontaneous resistant mutant; SNV, single nucleotide variant; Del, deletion; In, insertion.
(DOC)

S4 Table GO enrichment analysis of differentially expressed genes. (XLS)

S5 Table MIC values of SPI031 against ESKAPE pathogens. (DOCX)
