# Supplementary figures and images for: Elucidation of the Mode of Action of a New Antibacterial Compound Active against Staphylococcus aureus and Pseudomonas aeruginosa
Source: PLoS One. 2016 May 11;11(5):e0155139. doi: 10.1371/journal.pone.0155139 (PMC4864301; doi:10.1371/journal.pone.0155139)

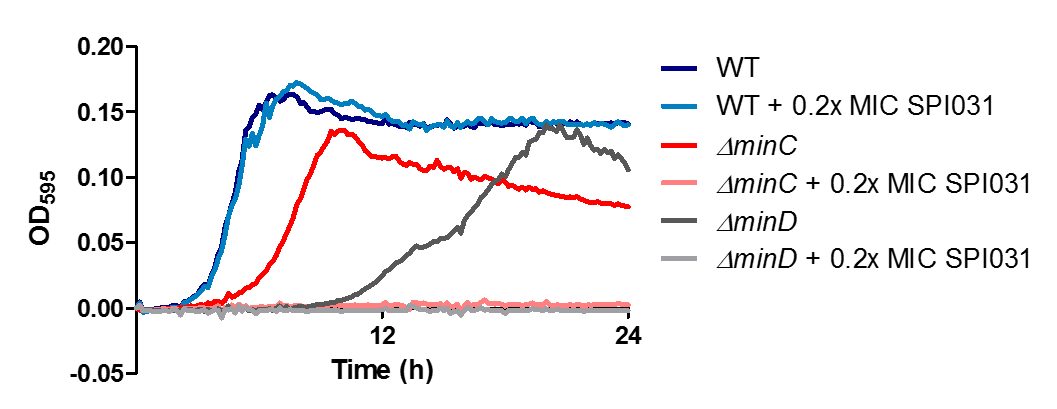

Supplement: S1 Fig — Growth curves of WT, minC mutant and minD mutant in the absence and presence of 0.2x MIC of SPI031. OD595 was measured every 10 min using an automated OD plate reader. The experiment was repeated three times and one representative repeat is shown. (TIF) [file pone.0155139.s001.tif]
