# Supplementary material for: Elucidation of the Mode of Action of a New Antibacterial Compound Active against Staphylococcus aureus and Pseudomonas aeruginosa
Source: PLoS One. 2016 May 11;11(5):e0155139. doi: 10.1371/journal.pone.0155139 (PMC4864301; doi:10.1371/journal.pone.0155139)
Supplement: S1 Table — (DOC) [file pone.0155139.s002.doc]

**Table S1: MIC values of SPI031 and other antibiotics against *S. aureus* and *P. aeruginosa***

| **Species** | **Antibiotic** | **MIC (µg/ml)** |
| --- | --- | --- |
| ***S. aureus*** | **SPI031** | 9.25 |
|  | **Ofloxacin** | 1 |
|  | **Tobramycin** | 0.0625 |
|  | **Vancomycin** | 0.125 |
|  | **Tetracycline** | 1 |
|  | **Ciprofloxacin** | 2 |
|  | **Rifampicin** | 0.008 |
|  | **Melittin** | 10 |
| ***P. aeruginosa*** | **SPI031** | 18.5 |
|  | **Ofloxacin** | 1 |
|  | **Tobramycin** | 0.0125 |
|  | **Polymyxin B** | 1 |
|  | **Melittin** | 10 |
