# Supplementary material for: Elucidation of the Mode of Action of a New Antibacterial Compound Active against Staphylococcus aureus and Pseudomonas aeruginosa
Source: PLoS One. 2016 May 11;11(5):e0155139. doi: 10.1371/journal.pone.0155139 (PMC4864301; doi:10.1371/journal.pone.0155139)
Supplement: S2 Table — (DOC) [file pone.0155139.s003.doc]

**Table S2: Bowtie2 alignment statistics for samples treated with DMSO (solvent control) and 0.2x MIC of SPI031**

| **Sample** | **Total reads** | **Reads aligned 0 times (%)** | **Reads aligned exactly 1 time (%)** |
| --- | --- | --- | --- |
| **DMSO 1** | 6303682 | 22.52 | 54.12 |
| **DMSO 2** | 9279873 | 32.86 | 31.72 |
| **DMSO 3** | 9477137 | 3.76 | 91.34 |
| **10 µM 1** | 9886738 | 14.69 | 24.99 |
| **10 µM 2** | 11936183 | 2.40 | 92.88 |
| **10 µM 3** | 10654739 | 2.04 | 93.23 |
