# Supplementary material for: Elucidation of the Mode of Action of a New Antibacterial Compound Active against Staphylococcus aureus and Pseudomonas aeruginosa
Source: PLoS One. 2016 May 11;11(5):e0155139. doi: 10.1371/journal.pone.0155139 (PMC4864301; doi:10.1371/journal.pone.0155139)
Supplement: S3 Table — 1Abbreviations: SRM, spontaneous resistant mutant; SNV, single nucleotide variant; Del, deletion; In, insertion. (DOC) [file pone.0155139.s004.doc]

**Table S3: Mutations identified in spontaneous SPI031-resistant mutants^1^**

|  | **Gene** | **Gene function** | **Mutation position** | **Type** | **Reference** | **Allele** |
| --- | --- | --- | --- | --- | --- | --- |
| **SRM1** | *htrB* | Lipid A biosynthesis | 1917117 | SNV | G | C |
|  | PA14_23400 | O-antigen biosynthesis | 2032149 | Del | G | - |
| **SRM2** | *nfxB* | negative regulator of MexCD-OprJ | 5428320-5428327 | Del | GGTATTCC | - |
|  | *nfxB* | negative regulator of MexCD-OprJ | 5428327-5428328 | In | - | T |
|  | - |  | 1647548 | Del | G | - |
| **SRM3** | *htrB* | Lipid A biosynthesis | 1917117 | SNV | G | C |
|  | - |  | 1375947 | SNV | A | G |
|  | - |  | 1647548 | Del | G | - |

^1^Abbreviations: SRM, spontaneous resistant mutant; SNV, single nucleotide variant; Del, deletion; In, insertion.
