# Supplementary material for: Elucidation of the Mode of Action of a New Antibacterial Compound Active against Staphylococcus aureus and Pseudomonas aeruginosa
Source: PLoS One. 2016 May 11;11(5):e0155139. doi: 10.1371/journal.pone.0155139 (PMC4864301; doi:10.1371/journal.pone.0155139)
Supplement: S5 Table — (DOCX) [file pone.0155139.s006.docx]

**Table S5: MIC values of SPI031 against ESKAPE pathogens**

| **Species** | **MIC (µg/ml)** |
| --- | --- |
| *Pseudomonas aeruginosa* PA14 | 18.5 |
| *Klebsiella pneumoniae* LMG 2095 | 4.63 |
| *Acinetobacter baumannii* NCTC 13423 | 18.5 |
| *Enterobacter aerogenes* LMG 2094 | 18.5 |
| *Enterococcus faecium* LMG 8148 | 9.25 |
| methicillin-resistant *Staphylococcus aureus* ATCC 33591 | 9.25 |
